# Phytochemicals: Target-Based Therapeutic Strategies for Diabetic Retinopathy

**DOI:** 10.3390/molecules23071519

**Published:** 2018-06-23

**Authors:** Amna Parveen, Jin Hyun Kim, Byeong Gyu Oh, Lalita Subedi, Zahra Khan, Sun Yeou Kim

**Affiliations:** 1 Department of Pharmacognosy, College of Pharmacy, Government College University Faisalabad, Faisalabad 3800, Pakistan; amnaparvin@gmail.com; 2 College of Pharmacy, Gachon University, Hambakmoero, Yeonsu-gu, Incheon 406-799, Korea; nirvana995@naver.com (J.H.K.); saxa12@naver.com (B.G.O.); subedilali@gmail.com (L.S.); Zahra.khan37@gmail.com (Z.H.); 3 Gachon Institute of Pharmaceutical Science, Gachon University, Hambakmoe-ro, Yeonsu-gu, Incheon 406-799, Korea

**Keywords:** diabetic retinopathy, protein kinase C, advanced glycation end-products, phytochemicals, oxidative stress, mitogen-activated protein kinases, vascular endothelial growth factor, poly (ADP-ribose) polymerase, metalloproteinase-9, reactive oxygen species, aldose reductase

## Abstract

*Background*: A variety of causative factors are involved in the initiation of diabetic retinopathy (DR). Current antidiabetic therapies are expensive and not easily accessible by the public. Furthermore, the use of multiple synthetic drugs leads to severe side effects, which worsen the diabetic patient’s condition. Medicinal plants and their derived phytochemicals are considered safe and effective treatment and their consumption can reduce the DR risk. In this article, we discuss a variety of medicinal plants, and their noteworthy bio-active constituents, that will be utilized as target based therapeutic strategies for DR. *Methods*: A broad-spectrum study was conducted using published English works in various electronic databases including Science Direct, PubMed, Scopus, and Google Scholar. *Results*: Targeting the multiple pathological factors including ROS, AGEs formation, hexosamine flux, PARP, PKC, and MAPK activation through variety of bioactive constituents in medicinal plants, diabetes progression can be delayed with improved loss of vision. *Conclusions*: Data reveals that traditional herbs and their prominent bioactive components control and normalize pathological cellular factors involved in DR progression. Therefore, studies should be carried out to explore the protective retinopathy effects of medicinal plants using experimental animal and humans models.

## 1. Introduction

Diabetes mellitus (DM) occurs due to an abnormality in blood glucose metabolism in which production or activity of insulin is adversely affected. The global prevalence of diabetes is rising rapidly and has serious socio-economic implications as it primarily affects the working-age population. According to a World Health Organization (WHO) report, the number of diabetic patients will have doubled from 171 million in 2000 to 366 million in 2030. In addition, the global prevalence of diabetes among adults older than 18 years has risen from 4.7% to 8.5% in the period from 1980 to 2014 [1]. This diabetic epidemic is of concern in that it does not only detriment patients’ health, but also is an economic burden on society as well as the patient. Increased risk of development of macro- and microvascular diseases including diabetic nephropathy, retinopathy, neuropathy, and of conditions affecting the cardiovascular system, is considered as the foremost causes of mortality among diabetic patients. Among the various diabetic complications, diabetic retinopathy (DR) is not only considered a serious microvascular complication of diabetes, and the main cause of decreased vision and blindness worldwide, but also has an additional neurodegenerative aspect. With the increasing prevalence of diabetes, the number of people affected by DR is expected to rise to 191 million by 2030 [2].

In diabetic retinopathy development, the microvasculature of the retina is damaged and its penetrability increased, which potentially results in loss of vision. Experimental evidence indicates that thinned retinas isolated from animal models exhibit a reduced number of retinal ganglion cells. Increased numbers of apoptotic cells within those retinas result in more rapid apoptosis before the appearance of histopathological manifestations [3].

Diabetes stimulates several factors and metabolites within the retina of diabetic animals that cause damage to retinal cells and affect disease progression. Oxidative stress (OS), which interacts with various signaling pathways, is considered the main factor for the initiation of the complex mechanism of diabetes [4]. DR is a complex mechanism interlinked with a number of dysregulated metabolic functions including inflammation, oxidative stress, increased formation of advanced glycation end-products (AGE), activation of protein kinases C (PKC), and stimulation of the polyol and hexosamine pathways [5]. Dysregulation of various metabolites within the diabetic retina causes a change in the production pattern of various mediators including growth factors, cytokines, chemokines, neurotrophic factors, and adhesion molecules. This results in increased oxidative stress, which exacerbates the retinal cells, triggering diabetic retinopathy [6].

Natural products offer a wide variety of therapeutically active compounds, which are considered less toxic, safer, and cheaper than synthetic compounds. These naturally derived compounds including alkaloids, tannins, polyphenols, terpenoids, flavonoids, and steroids exhibit different pharmacological effects by simultaneously targeting multiple metabolic pathways [7,8,9]. In addition, these naturally derived chemicals also contribute to improved intercellular drug delivery, which is considered an important factor in attaining good therapeutic effects [10,11]. Using modern natural research techniques, many potentially active compounds have been discovered and utilized to ameliorate the DR. This review highlights recently-studied agents derived from natural products with respect to their role in the treatment of diabetes-related complications, and which may be a source of alternate therapies against retinal damage caused by diabetes.

## 2. Results

### 2.1. Signaling Pathway of Diabetic Retinopathy

In diabetic retinopathy, a multifactorial disease, retinal cells produce a variety of cytokines, growth factors, and enzymes, which are interlinked and are believed to initiate the cellular mechanisms underlying the condition [12]. The development of DR requires a variety of mechanisms including oxidative stress [6], PKC activation, the formation of AGEs, hexosamine flux, poly (ADP-ribose) polymerase (PARP) activation, and mitogen-activated protein kinase (MAPK) activation, which cause faster apoptosis of retinal cells in diabetes [5] and ultimately lead to the development of DR.

Various substances interact with each other to drive Janus kinase/signal transducers and activate transcription of the JAK/STAT pathway, which plays a significant role in retinal diseases. Receptor tyrosine kinase 2 (TYK2), JAK1, JAK2, and JAK3 belong to the JAK class of tyrosine kinases while STAT-1, -2, -3, -4, -5a, 5b, and 6b are members of the signal transducer and activator of transcription (STAT) family [13]. Interferon-gamma (INF-γ) triggers JAK1 and JAK2 phosphorylation through receptor binding, and activates STAT-1 through its tyrosine residue, followed by dimerization of STAT1 molecules and, together with interferon regulatory factor 1 (IRF-1), translocate into the nucleus and forms a complex. This complex activates interferon-stimulated response element (ISRE), which is followed by cytokines production [14]. Cytokines production can activate ROS production and vice versa, suggesting a bidirectional mechanism. Increased ROS results in retinal cell injury through interaction with different cellular components, and ultimately increases the incidence of DR [4].

The interaction between AGE and its receptors (AGE-RAGE) results in activation of various signaling pathways including those involving nicotinamide adenine dinucleotide phosphate (NADPH) oxidase and reactive oxygen species (ROS), MAP kinase, p38 MAPK, RAC, cell division control protein 42 (CDC42), and p21 RAS expression. This causes nuclear factor kappa b (NF-*κ*B) activation followed by elevation of transcriptional protein, the inflammatory response including apoptosis, pro-inflammatory gene expression, and migration, ultimately aggravating DR [5,15].

Intracellular hyperglycemia elevates de novo synthesis of diacylglycerol (DAG), increased levels of which, in turn, activate several kinase isoforms from the PKC family, and control abnormalities in retinal and renal blood flow in diabetic mice. Activation of PKC induces vascular endothelial growth factor (VEGF) and transforming growth factor beta 1 (TGF-β1) expression [5], followed by accumulation of fibronectin and type IV collagen in glomeruli of diabetic rats. All of these changes ultimately contributed to changes in the microvascular protein matrix [16]. Furthermore, PKC is also involved in controlling these changes by inhibiting the nitric oxide production. In addition, PKC activation contributes to NF-κB activation, plasminogen activator-1 (PAI-1) overexpression, and to stimulation of NADPH dependent oxidases. Therefore, treating diabetic mice with PKC inhibitors can reduce PKC activity and ultimately control hyperglycemia, providing protection against abnormalities related to DR [5,17].

Several diabetic complications may result from the shunting of excess intracellular glucose into the hexosamine pathway. Elevated levels of substances involved in the hexosamine signaling pathway have been found in retinal tissues from rats and humans with diabetes. During glycolysis, fructose-6-phosphate triggers glucosamine-mediated activation of the PAI-1 promotor via serine protease 1 (SP1) sites in glomerular mesangial cells. Modification of SP1 through *N*-acetylglucosamine may explain the relationship between activation of the hexosamine pathway and hyperglycemia. Additionally, the hexosamine pathway regulates the PAI-1 gene promoter, as well as SP1 transcriptional gene activation via PKC-β1 and -γ and other transcription factors which play an important role in the pathogenesis of diabetic complications [5,18].

The polyol pathway, a key component in glucose metabolism, plays a pivotal role in the pathogenesis of cataract formation, refractive changes, and diabetic retinopathy in diabetic individuals. High expression of aldose reductase, a key rate-limiting enzyme in polyol pathway, reduces the conversion of glucose and galactose into sorbitol and galactitol. Excess sorbitol lowers available NADPH levels, which results in elevated oxidative stress, ultimately resulting in tissue retinal damage, aggravating DR [19,20]. Therefore, DR can be ameliorated through mediation and control of the polyol pathway.

PARP, a nuclear enzyme believed to be active in the retina of diabetic animals, causes DNA damage and exacerbates nitrosative and oxidative stress, potentially activating NF-κB and its dependent genes intercellular adhesion molecule (ICAM), monocyte chemoattractant protein-1 (MCP-1), and tumor necrosis factor alpha (TNF-α). Inhibition of glyceraldehyde-3-phosphate dehydrogenase activity by PARP activates PKC, the hexosamine pathway, and AGE formation, triggering ROS production, thereby playing an important role in the pathogenesis of DR [21]. Therefore, by blocking PARP activity, PKC activation, the hexosamine pathway, and AGE formation associated with DR can be blocked or attenuated.

In diabetes, metalloproteinase-9 (MMP-9) is activated. MMP-9 regulation is controlled by various transcriptional factors, including NF-κB and activator protein 1 (AP-1). PARP-1, a well-characterized member of PARP family, triggers and regulates the formation of complexes between these transcriptional factors with MMP-9. All these changes lead to increase the expression of MMP-9 causing mitochondrial damage and capillary cell apoptosis. Therefore, through inhibition of PARP-1 activity, retinopathy can be prevented in diabetic individuals [22].

**Figure 1:**
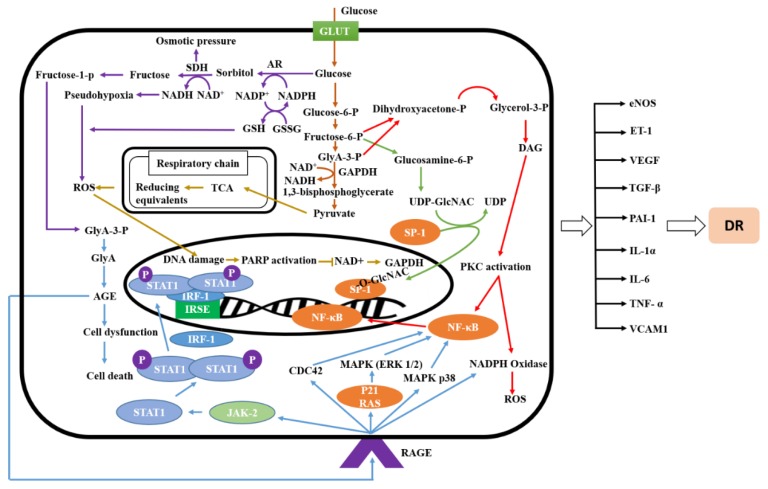
Signaling pathways involved in DR. eNOS, Endothelial nitric oxide synthase; ET-1, Endothelin-1; VEGF, Vascular endothelial growth factor; TGF-β1, Transforming growth factor beta; PAI-1, Plasminogen activator inhibitor-1; IL- Interleukin; TNF-α, Tumor necrosis factor alpha; VACM-1, Vascular cell adhesion molecules-1; DAG, Diacylglycerol; PKC, Protein kinase C; NF-κB, Nuclear factor kappa; ROS, Reactive oxygen species; MAPK, Mitogen-activated protein kinase; RAGE; Receptor for AGE; CDC42, Cell division control protein 42; UDP-GlcNAC, Uridine diphosphate *N*-acetylglucosamine; AR, Aldose reductase; TCA, Tricarboxylic acid; STAT1, Signal transducer and activator of transcription 1; IRF-1; Interferon regulated factor 1; PARP; Poly (ADP-ribose) polymerase; GLUT, Glucose transporter; RAS, Renin-angiotensin system.

ROS, PKC activation, AGEs production, hexosamine flux, PARP activation, and MAPK activation are interlinked to each other, and are the main causative agents in the pathogenesis of DR. Inhibition of the mechanisms described above can prevent the apoptosis of retinal cells in diabetes, and ultimately delay and ameliorate DR as shown in Figure 1.

### 2.2. Anti-diabetic Effects of Phenolic and Flavonoid Phytochemicals

#### 2.2.1. *Abeliophyllum distichum*

**Figure 2:**
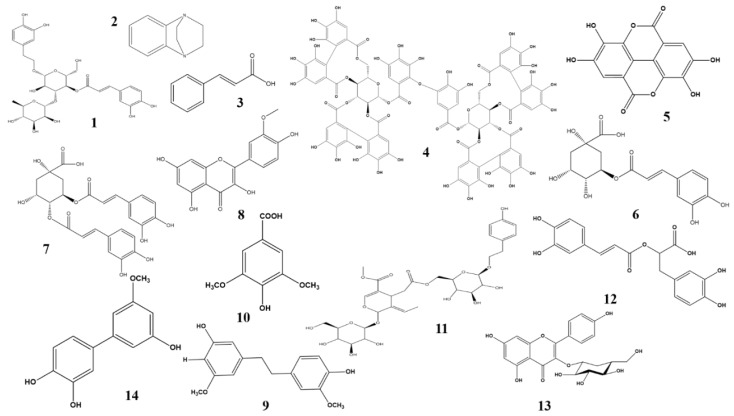
**1**. Acetoside—*Abeliophyllum distichum*; **2**. benzo[*b*]-1,4-diazabicyclo[2.2.2]octane—*Aegle marmelos*; **3**. Cinnamic acid—*Aegle marmelos*; **4**. Agrimoniin—*Agrimonia pilosa*; **5**. Ellagic acid—*Carpobrotus edulis*; **6**. Chlorogenic acid—*Aster Koraiensis*; **7**. 3,5-dicaffeoylquinic acid—*Aster koraiensis*; **8**. Isorhamnetin—*Cochlospermum religiosum*; **9**. Gigantol—*Dendrobium chrysotoxum*; **10**. Syringic acid—*Dendrobium chrysotoxum*; **11**. Specnuezhenide—*Ligustrum lucidum*; **12**. Rosmarinic acid—*Melissa officinalis*; **13**. Astragalin—*Moringa oleifera*; **14**. 5′-methoxybiphenyl-3,4,3′-triol—*Osteomeles schwerinae*.

*A. distichum*, also called Korean abeliophyllum or white forsythia, is a monotypic genus of flowering plants. *A. distichum* Nakai is endemic to the southern and central regions of Korea. Major isolated compounds are quinol glycosides, acteoside, and halleridone. These have a potent inhibitory effect on aldose reductase. Acetoside (Figure 2), the most potent ingredient, has a five times greater efficacy against aldose reductase than quercetin [23].

#### 2.2.2. *Aegle marmelos*

**Figure 3:**
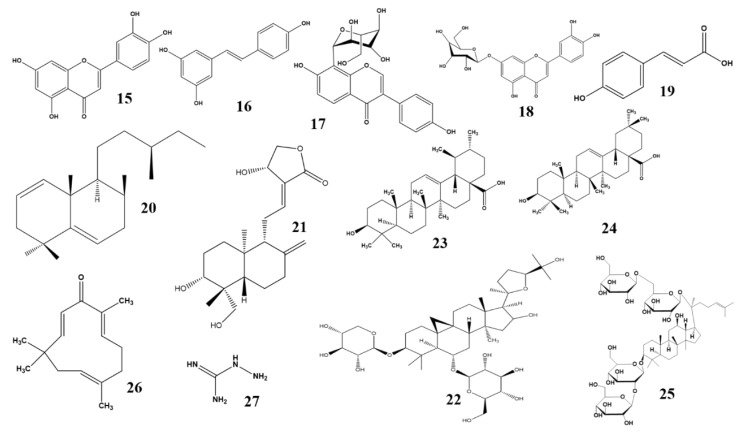
**15**. Luteolin—*Platycodon grandiflorum*; **16**. Resveratrol—*Polygonum cuspidatum*; **17**. Puerarin—*Puerariae lobate*; **18**. luteolin-7-glucoside—*Vitex negundo*; **19**. Coumaric acid—*Zea mays*; **20**. Labdadiene—*Alpinia zerumbet*; **21**. Andrographolide—*Andrographis paniculata*; **22**. Astragaloside IV—*Astragalus membranaceous*; **23**. Ursolic acid—*Origanum majorana* L.; **24**. Oleanolic acid—*Origanum majorana* L.; **25**. Ginsenoside-Rb1—*Panax quinquefolius*; **26**. Zerumbone—*Zingiber zerumbet*; **27**. Aminoguanidine—*Paenonia lactiflora.*

*A. marmelos*, commonly called Bael, is distributed across several regions of India and South East Asia. Evidence from in vitro, ex vivo, and in vivo experiments suggest that it has the potential to inhibit aldose reductase in rat lens, which is associated with lens opacification. Greater chaperone activity was reported upon treatment with α-crystalline, a water-soluble protein isolated from rat’s lens, with ethyl acetate extract. Phytochemical profiling showed the presence of three major compounds, namely benzo[*b*])-1,4-diazabicyclo[2.2.2]octane (Figure 2), cinnamic acid (Figure 2), and 3,4-dimethoxybenzoic anhydride. These were isolated from an ethyl acetate extract but, while these compounds may be responsible for ameliorating DR, activity assays involving these compounds have not yet been performed [24].

#### 2.2.3. *Agrimonia pilosa* Ledeb

*A. pilosa*, is well known in traditional Chinese medicine. The butanol fraction of a methanol extract of this plant was found to be a rich source of flavonoids and contained bioactive compounds including agrimoniin (Figure 2), quercetin, afzelin, luteolin, and luteolin-7-glucoside. These exhibited significant inhibitory activity against aldose reductase [25]. A triterpenoid compound isolated from this plant helps in preadipocyte-differentiation via peroxisome proliferator-activated receptor gamma (PPAR-γ) activation. In addition, it can potentially activate the mRNA expression of adiponectin and glucose transporter type 4 (GLUT4) [26].

#### 2.2.4. *Aster koraiensis*

*A. koraiensis*, a valuable Korean native plant, is employed in traditional medicine and as a food in Korea. Kim et al. demonstrated in 2016 that *A. koraiensis* extract attenuates retinal pericyte apoptosis by suppressing AGE formation and NF-κB activation. Chlorogenic acid (Figure 2) and 3,5-dicaffeoylquinic acid (Figure 2) are the two major bioactive constituents responsible for this activity in this plant [27]. Further studies have indicated that this plant can also prevent retinal vascular dysfunction, block blood-retinal barrier (BRB) breakage, and inhibit tight junction protein expression. This highlights the potential benefits of *A. koraiensis* as a dietary supplement for attenuating DR.

#### 2.2.5. *Camellia nitidissima* Chi

*C. nitidissima* Chi, an edible plant, is used as a tea in China. Usually, it grows in a narrow region of Northern Vietnam and Southern China. This plant is a good source of flavonoids and glycosides and exhibits beneficial properties including anti-AGE formation activity and methylglyoxal scavenging. This suggests that it can reduce diabetic complications, including DR, via anti-AGE formation and anti-oxidant mechanisms [28]. However, additional studies need to be carried out to characterize its bioactive phytochemicals and to elucidate their anti-AGE formation mechanism and preventive role in DR.

#### 2.2.6. *Carpobrotus edulis*

*C. edulis* is widely distributed in the Eastern Cape of South Africa. This plant contains ferulic acid and ellagic acid (Figure 2), which exhibit the attenuative properties against DR via inhibition of AGE formation, and of OS [29]. Recently, a study conducted to note the potency of ellagic acid revealed that it is involved in the prevention of retinal abnormalities through inhibition of AGE formation. A detailed mechanism indicates that ellagic acid inhibits the expression of GAP, VEGF, Bax, and hypoxia-inducible factor 1-alpha (HIF-1α), and apoptosis in the retina [30].

#### 2.2.7. *Cochlospermum religiosum*

*C. religiosum*, a small or medium-sized deciduous tree, is commonly known as “Golden silk cotton”. Isorhamnetin (Figure 2), a flavonoid glycoside, present in this plant exhibited an anti-oxidant effect by suppressing lipoprotein diene formation induced by Cu^2+^, and provided a protective effect against cataracts induced by selenite in an in vitro cell culture model. The anti-oxidant effect of this compound makes this plant valuable in the treatment of DR [31,32]. In vitro and in vivo studies have revealed that myricetin, a bioactive flavonoid, is active against DR through inhibition of RAGE-Src-ERK1/2-FAK-1 paxillin signaling pathway, followed by prevention of retinal pericyte migration [28].

#### 2.2.8. *Dendrobium chrysotoxum* L.

In 2014, Wang et al. described the potency of an ethanolic extract of *D. chrysotoxum* against DR and its mechanism. The experimental results indicated that the extract decreases elevated retinal vessels in diabetic rats [33]. In addition, it lowered the increased expression of VEGF and VEGFR2 mRNA, as well as serum VEGF levels. In treated diabetic rats, the levels of retinal MMP2/9, serum MMP 2/9, platelet-derived growth factor (PDGF)A/B, basic fibroblast growth factor, insulin-like growth factor, IL-1β, IL-6, p65 phosphorylation, and ICAM-1 were suppressed. These findings demonstrate the potency of *D. chrysotoxum* in reversing DR [34]. Further experiments on the potency of *D. chrysotoxum* showed that it attenuates the breakdown of the blood-retinal barrier. It also reverses the decreased expression of retinal mRNA for the junction proteins occludin and claudin-1 in diabetic rats. Furthermore, it reduces ICAM-1 expression, TNFα, IL-6, IL-12, IL-3, IL-2, IL-10, IκB, and IκB kinases. Together, this reveals that this plant reduces DR by preventing retinal inflammation and reducing the amount of tight junction protein in diabetic rats. It can, therefore, be recommended as a supplementary treatment for diabetic patients [35]. Further studies on its bioactive constituents demonstrated that gigantol (Figure 2), an important bioactive constituent, has a synergistic effect with syringic acid (Figure 2), and plays an important role in preventing diabetic cataracts by inhibiting aldose reductase activity through downregulation of its expression via reduced transcription, and by controlling sorbitol levels. The synergistic effect is thus mainly due to disruption of AR activity and the polyol pathway [36].

#### 2.2.9. *Ginkgo biloba*

*G. biloba* is commonly known as maiden hair. A combined extract of red berry, white willow bark, and *G. biloba* was evaluated against DR, with the experimental results indicating lowered TNF-α and VEGF expression, thereby attenuating elevated plasma lipid peroxidation and retinal inflammation in diabetic rats [37]. Extracts of this plant have been found to notably improve retinal capillary blood flow rate in patients with diabetic retinopathy [38]. In addition, clinical trials on the effectiveness of *G. biloba* extract (GBE) in patients with DR showed that GBE prevents and suppresses microvascular alterations in the retinas of diabetic patients, and ultimately improves DR [39]. Recently, a study demonstrated that GBE significantly decreases the number of retinal microaneurysms and areas of retinal hemorrhage, and also decreases in total cholesterol and low-density lipoprotein. In addition, decreases in the rate of platelet aggregation and adhesion in diabetic patients upon treatment with GBE proves its efficacy against DR [40]. Several clinical trials have now demonstrated that GBE may be used as a first-line therapy against DR.

#### 2.2.10. *Glycyrrhiza uralensi*

*G. uralensi* is used as a natural sweetener. Many studies have suggested a potential role of *G. uralensi* in improving glucose tolerance through the PPAR-γ pathway, due to the presence of bioactive compounds such as glycycoumarin, glycyrin, and dihydroglyasperin C and D. Additionally, semilicoisoflavone B, liquiritigenin, and isoliquiritigenin have, through their effects on DR and sorbitol formation, indicated the plant’s potential therapeutic importance against diabetes and diabetes-related complications [41].

#### 2.2.11. *Juglans regia* L.

*J. regia* L. has hypoglycemic effects of *J. regia* L. that indicates the value of this plant in significantly lowering the blood glucose during fasting and HbA1c. Moreover, leaf extracts from this plant attenuate lipid peroxidation, anti-oxidant status, S100B, PARP, cyclooxygenase 2 (COX-2), and caspase-3 expression in the retina of diabetic rats [42]. *J. regia* L. is a rich source of flavonoids and phenolic compounds that might be related to its potency against DR.

#### 2.2.12. *Litchi chinensis*

*L. chinensis* is commonly known as lychee. Its natural range includes Vietnam, China, and the Malay Peninsula. The plant is a rich source of anthocyanins, phenolics, and flavonoids which demonstrate diverse pharmacological activities including anti-cancer, anti-oxidant, anti-coagulant, immunomodulatory, and liver-protective effects. Extracts of the fruit pericarp, which are under consideration as additives in the food and pharmaceutical industries, have inhibitory effects against glycoxidation and AGE formation in serum and lens homogenate. *L. chinenesis* also inhibits changes in fundus structure and protects retinal cells in streptozotocin (STZ)-induced diabetic rats. These findings demonstrate that *L. chinenesis* delays the progression and development of DR by inhibiting oxidative stress, glycoxidation and AGE formation due to its high polyphenol and tannin content [43].

#### 2.2.13. *Ligustrum lucidum* Ait

*L. lucidum* is a well-known traditional Chinese medicine possessing a wide range of pharmacological functions. Specnuezhenide (Figure 2), a constituent of this plant, is valued for its effectiveness against DR through suppression of the HIF-1α/VEGF signaling pathway. Specnuezhenide suppresses VEGF expression, downregulating mRNA expression of VEGFA, prolyl-hydroxylase 2, and HIF-1α. In addition, it prevents hypoxia-induced retinal neovascularization in a rat model and prevents DR. This indicates that specnuezhenide can potentially be used to treat DR [44].

#### 2.2.14. *Lonicerae japonicae* Flos

*L. japonicae* Flos decreases retinal angiogenesis in diabetic mice and reduces VEGF-induced tube formation owing to the presence of chlorogenic acid, caffeic acid, and luteolin [45]. Chlorogenic acid is considered to be the main compound involved in suppression of retinal angiogenesis and works by decreasing VEGF expression, which is followed by a decreased breakdown of BRB, and of vascular leakage. This provides a protective effect against DR production in diabetic rats [46]. Further studies have indicated that it lowered VEGF expression and VEGF-mediated retinal neoangiogenesis in STZ-induced hyperglycemic mice. Collectively, these results highlight the potency of chlorogenic acid contained in *L. japonicae* against DR [47].

#### 2.2.15. *Melissa officinalis*

*M. officinalis* anti-oxidant property has been studied extensively, and found to be principally responsible for lowering hyperglycemia and AGE formation. *M. officinalis* extracts have the ability to elude the cross-β structure pathway during glycation. Research involving alloxan-induced diabetic rats revealed that *M. officinalis* significantly suppresses diabetes by modulating blood sugar, lipoprotein, and serum lipid levels. In addition, it has a beneficial effect on high-density lipoprotein (HDL) levels [48]. The plant, therefore, has potential therapeutic uses in the treatment and prevention of diabetes and related disorders [49]. Rosmarinic acid (Figure 2), a major phenolic bioactive compound isolated from this plant, has a potential role in suppressing DR. It inhibits the proliferation of retinal endothelial cells and inhibits angiogenic tube formation. According to a study using a mouse model, rosmarinic acid’s suppression of retinal neovascularization is linked to cell cycle arrest followed by an increase in p21 (WAF1) expression. Interestingly, rosmarinic acid does not exhibit any retinal toxicity [50]. It is clear that *M. officinalis*-derived rosmarinic acid acts as a potent inhibitor of retinal neovascularization. However, the details of the DR-linked signaling mechanism still need to be explored.

#### 2.2.16. *Moringa oleifera* Lam

*M. oleifera* Lam is mainly consumed in Pakistan, India, Hawaii, and across Africa. Polyphenolic compounds from *M. oleifera* are responsible for lowering blood glucose by stimulating glucose uptake. In the diabetic retina, an increased expression of TNF-α, IL-1β, VEGF, PKC-β was observed. On treating the diabetic retinae with *M. olefera*, a marked inhibition in these expressions was noted. In addition, *M. oleifera*-treated retinae exhibited intact retinal vasculature and reduced thickening of the capillary basement membrane. This was evidence of *M. oleifera*’s efficacy against diabetic retinopathy via anti-oxidant, anti-inflammatory, and anti-angiogenic mechanisms [51]. Astragalin (Figure 2), a major bioactive compound present in this plant, has been evaluated as an anti-diabetic, and was found to both lower VEGF expression and to inhibit the negative effects of high glucose levels, effectively preventing DR [52].

#### 2.2.17. *Morus alba*

*M. alba* is commonly known as white mulberry and is broadly cultivated in subtropical, tropical, and moderate regions. Owing to the presence of flavonoids, coumarins, and phenols, the leaves of this plant possess a variety of anti-hypotensive, anti-hyperglycemic, and neuroprotective properties. Recently, it has been demonstrated that oral administration of *M. albla* potentially reduces glucose levels and suppresses the production of sorbitol, PKC, OS, and proinflammatory cytokines in retinas of diabetic rats. Moreover, it also downregulates caspase-3 and Bax and upregulates B-cell lymphoma 2 (Bcl-2). In addition, it also inhibits the VEGF expression in the retina of diabetic rats. Collectively, these findings suggest that *M. albla* exerts its protective effect against DR by inhibiting hyperglycemia-induced apoptosis, inflammation, polyol pathway activation, oxidative stress, and VEGF expression [53].

#### 2.2.18. *Osteomeles schwerinae* C.K. Schneid

*O. schwerinae* C.K. Schneid ethanolic extract contains significant quantities of quercitrin and hyperoside, which exhibit inhibitory properties against rat aldose reductase. An additional potentially-bioactive constituent is 5′-methoxybiphenyl-3,4,3′-triol (Figure 2), which also exhibited inhibitory activity against aldose reductase. Further study revealed that it interrupts AGE formation, reduces VEGF expression, causes breakdown of the blood-retinal barrier, and stabilizes the junction protein occludin. It also downregulates the various angiogenic factors including fibroblast growth factor (FGF-2), insulin-like growth factor-binding proteins (IGFBPs), and PAI-1. All these unveils the potency of 5′-methoxybiphenyl-3,4,3′-triol against DR pathogenesis via an anti-VEGF mechanism and the hexosamine pathway [33]. Further study, exploring the potency of this compound against DR via different signaling pathways, is warranted. In addition, in order to improve sample quality, standardization of *O. schwerinae* extractions using 5′-methoxybiphenyl-3,4,3′-triol as a marker bioactive compound should be undertaken.

#### 2.2.19. *Perilla frutescens*

*P. frutescens*, commonly known as perilla or Korean perilla. It is an annual short-day plant. Rosmarinic acid, a potent bioactive compound present in ethyl acetate fractions of methanol extracts of *P. frutescens*, exhibited aldose reductase inhibitory activity. [54]. In a prior study, it is indicated that rosmarinic acid suppresses the proliferation of retinal endothelial cells and prevents retinal neovascularization through cell cycle arrest by increasing expression of p21WAF1. [50].

#### 2.2.20. *Platycodon grandiflorum*

The potency of *P. grandiflorum* against diabetes is due to the presence of luteolin (Figure 3), luteolin glucoside, luteolin acetyl glucoside, and chlorogenic acid methyl ester, which, isolated from this plant, proved to be potent inhibitors of AGE formation. Luteolin glucoside exhibited a potent inhibitory effect against rat lens aldose reductase (RLAR) activity [55]. Recently, the clinical effect of a herbal decoction containing different medicinal plants, including *P. grandiflorum*, was studied in patients with stage II DR, with the finding that it effectively prevented and treated DR. Clinical reports has proven that supplementation of DR patients with *P. grandiflorum* could be an effective therapy for DR [56].

#### 2.2.21. *Polygonatum odoratum*

*P. odoratum* contain three homoisolflavones, namely 3-(4′-hydroxybenzyl)-5,7-dihydroxy-6-methyl-8-methoxychroman-4-one, 3-(4′-methoxybenzyl)-5,7-dihydroxy-6-methyl-8-methoxychroman-4-one, and 3-(4′-hydroxybenzyl)-5,7-dihydroxy-6,8-dimethylchroman-4-one, are isolated from the chloroform fraction of the ethanol extract of this plant’s rhizome. These compounds exhibit inhibitory activity against AGE formation in both in-vitro and in-vivo studies [57].

#### 2.2.22. *Polygonum cuspidatum*

*P. cuspidatum* is used in folk medicines from China, Japan, and Korea, as it possesses a variety of biological activities against allergies, diabetes, and as an anti-viral agent. Kim et al. (2012) demonstrated that *P. cuspidatum* can ameliorate HMGB1, RAGE, and NF-κB activities in the retina. In addition, it decreases retinal vascular permeability and the loosening of tight junctions in diabetic rats [35]. This plant can, therefore, be recommended as an effective supplement for the treatment of DR. Additional research into the effectiveness of P. *cuspidatum* indicated that it controls RAGE-mediated activation of NF-κB, followed by inhibition of upregulation of HMGB1 and prevention of diabetes-induced retinal vascular hyperpermeability [58]. *P. cuspidatum* is known to contain four major active compounds: polydatin, resveratrol (Figure 3), emodin glucopyranoside and emodin. It has been shown that resveratrol provides protection against ADR via inhibition of the AKT/NF-κB signaling pathway as well as through suppression of high morbidity group box 1 (HMBG1), RAGE, TNF-α, and IL-4 in skin inflammation induced by atopic dermatitis. Emodin also suppresses HMGB1, NF-κB, and gene expression of cell surface adhesion proteins. Emodin glucopyranoside, on the other hand, has the capacity to function as an antioxidant [58]. Recently, it has been demonstrated that polydatin provides it hepatoprotective effect through the peroxisome proliferator-activated receptor alpha/beta (PPAR-α/-β) signaling pathway in STZ induced diabetic mice [59]. Collectively, these bioactive compounds allow *P. cuspidatum* to exert its effects against DR.

#### 2.2.23. *Polygonum multiflorum*

*P. multiflorum* is still popular in both China and Japan, but is now also popular in North America. Tetrahydroxystilbine glucoside, a major bioactive compound in this plant, inhibits AGE formation by trapping reactive methylglyoxal under physiological conditions in a dose-dependent manner [60] (Lv, Shao et al. 2010). These results indicate that it has the capability to delay DR.

#### 2.2.24. *Prunella vulgaris*

*P. vulgaris*, a self-healing herb, is broadly distributed across Asia, Europe, North America, and Northwest Africa. It contains several bioactive compounds, including oleanolic acid, ursolic acid, botulinic acid, rosmarinic acid, caffeic acid ethyl ester, 2-hydroxy cinnamic acid, and prunellin. Caffeic acid ethyl ester, p-hydroxy cinnamic acid, and prunellin are the main active compounds responsible for inhibition of RLAR activity. Furthermore, caffeic acid ethyl ester ameliorates diabetic retinopathy via an anti-oxidant, anti-AGE formation mechanism, and has been shown to possess stronger activity than the commonly-prescribed drug aminoguanidine. These findings suggest that *Prunella vulgaris* is a potential source of active components capable of ameliorating DR [61]. However, as is the case with many other medicinal plants, additional studies using animal models are needed to unveil the mechanisms underlying its anti-AGE formation activities.

#### 2.2.25. *Pueraria lobata*

*P. lobate* contains bioactive isoflavonoid puerarin (Figure 3), which is isolated from its dried root. Puerarin prevents retinal and neuronal cells from undergoing apoptosis via an anti-oxidant mechanism, and possesses a number of additional activities. It reduces the mRNA expression of inducible nitric oxide synthase (iNOS), increases SOD activity, and inhibits radical formation. It reduces oxidative stress by downregulating RAGE expression and prevents assembly of the receptor complex. It also suppresses NF-κB and NADPH oxidase activation via suppression of p47phox and Rac1 signaling and prevents apoptosis in retinal pericytes. Additionally, it exhibits anti-angiogenic properties by suppressing the expression of HIF-1α and VEGF mRNA. Finally, it exhibits its anti-inflammatory effect by attenuating IL-1β, ICAM, VCAM-1, cell apoptosis, Bax, and Caspase-3 while simultaneously upregulating Bcl-2 expression in mitochondria, and blocking BRB breakdown, ultimately preventing DR. Genistein, a bioactive flavonoid, attenuates diabetic retinopathy by inhibiting tyrosine kinase, proinflammatory cytokine production, TNF-α activation, and microglial activation. It prevents leukocytes/endothelial interaction and vascular dysfunction and inhibits extracellular-signal-regulated kinase (ERK) and p38 MAPK phosphorylation, ultimately preventing retinal edema. An isoflavone also present in this plant, daidzein, exerts its anti-inflammatory effect by acting on the PPAR receptor, providing protection against diabetic retinopathy [62]. Puerarin, *P. lobata*’s principal isoflavone glycoside, has been isolated from the plant and, upon evaluation, has been found to modulate VEGF expression by suppressing hypoxia-inducible factor-1 mRNA, ultimately exerting a protective effect against DR in experimental animals [63]. Additionally, it has been found to inhibit AGE formation. NADPH oxidase consists of membrane-integrated cytochrome b 558, which comprises several regulatory subunits that are required for the activation of AGE-BSA-induced NADPH oxidase and ROS production. Puerarin blocks the activation of AGE-BSA-induced phosphorylation of p47phox and Rac1, thus achieving the inhibitory effect. AGE-BSA also stimulates the translocation of NF-*κ*B, also causing hyperglycemia. Therefore, treatment with puerarin inhibits the AGE-BSA-induced NF-κB activity, and ultimately provides protection from diabetes and diabetes-related disorders [64]. These factors combined indicate that *P. lobata* is a potential source of flavonoid which highly-effective against DR. Animal trials indicate that this plant may form the basis for promising, effective medicines for use in first-line DR treatment therapies.

#### 2.2.26. *Salvia miltiorrhiza* Bge

*S. miltiorrhiza* Bge contains several active compounds which exhibit anti-diabetic effects, including isosalvianolic acid C methyl ester, tanshinone IIA, rosmarinic acid, salvianolic acid, and lithospermic acid dimethyl ester are present in this plant. Injection of *S. miltiorrhiza* extracts into the retinal hypoxic-ischemic tissues of DR mice promotes blood oxygen transport and retinal hemangioma absorption, while also controlling blood sugar levels and improving microcirculation. *S. miltiorrhiza* may prove an effective treatment for DR by way of the blood-ocular barrier, as it protects cells from damage and contributes to visual improvements [65]. Controlled clinical studies have been conducted to evaluate the effectiveness and safety of *S. miltiorrhiza* and have proven its effectiveness intreating DR [66,67]. Further studies indicate that salvianolic acid decreases the morbidity rate of cataract and improves the retinal pathological changes by preventing the chronic inflammation, inhibiting lipid peroxidation, and reducing the lipoprotein-related phospholipase A2 (Lp-PLA2) through down-regulating the IL-6, IL-1, oxidized low-density lipoprotein, and (Lp-PLA2) level [68].

#### 2.2.27. *Stauntonia hexaphylla*

*S. hexaphylla* (Thunb) Decne is broadly distributed in the lowlands and foothills of mostly warmer areas of Korea, Japan, and China, where it occurs as dense groups of bushes. The known chemical constituents of *S. hexaphylla* include glucosides, flavonoids, triterpenoids, phenolic glucosides, phenylpropanoids and chlorogenic acid analogs. Chlorogenic acid, quercetin-3-*O*-𝛽-d-glucopyranoside, luteolin-7-*O*-𝛽-d glucopyranoside, and calceolarioside B exhibit activity against RLAR. Additionally, luteolin-7-*O*-𝛽-d glucopyranoside, 3-(3-(3,4-dihydroxyphenyl)-propionic acid, and neochlorogenic acid showed anti-AGE formation activity. These results suggest that *S. hexaphylla* extracts have potential application in the treatment of cataracts caused by hyperglycemia and DR [69].

#### 2.2.28. *Tephrosia purpurea*

*T. purpurea* is widely cultivated for its anti-diabetic properties, which are contained within a flavonoid-rich alcoholic fraction of extractions. The mechanism underlying this indicates that it is involved in increasing the level of soluble proteins that prevent the cross-linking, aggregation and distribution of the soluble protein. It also possesses antioxidant properties that prevent insolubilization of proteins, delaying development of lens opacity. In addition to this, results from docking experiments have suggested that most of the known constituents including rutin, quercetin, lupeol, fidarestat, tephrosin degurelin rotenone, and elliptone have a common binding mode in the vicinity of the active site of subunit A of aldose reductase, lying between the catalytic amino acid residues, Trp20, Tyr48, Trp111, Phe122, and His110. *T. purpurea* possesses significant anti-hyperglycemic activity, as well as antioxidant activity in diabetic rats and has shown significantly in vitro AR inhibiting activity. Together with the ability to reduce oxidative stress and inhibition of AR, *T. purpurea* might be beneficial not only in preventing hyperglycemia but also in delaying the onset of diabetes-induced complications resulting from hyperglycemia-induced oxidative and osmotic stress [70].

#### 2.2.29. *Terminalia catappa*

*T. catappa* grows in the warmer regions of India. *T. catappa* is a rich source of tannin, which is linked to its anti-diabetic effect. The bark extract of *T. catappa* induced improvement in body weight and lipid profile along with regeneration of pancreatic β-cells in STZ-induced diabetic rats. Research into the effectiveness of *T. catappa* has indicated that it is rich in phytochemicals which have the capacity to reverse a number of pathologies induced by hyperglycemia. These include suppression of mitochondrial dysfunction, inhibition of superoxide generation, downregulation of inflammatory and angiogenic factors, restoration of balance between low-density lipoprotein (LDL) and high-density lipoprotein (HDL) cholesterol, and suppression of enzymatic-activity-related glucose absorption. *T. catappa* is thus valuable as an effective natural therapy to inhibit the progression of DR and has potential application in first-line therapies [71].

#### 2.2.30. *Vitex negundo*

*V. negundo*, commonly known as the five-leafed chaste tree, is famous for its diverse pharmacological properties. It is widely used in traditional medicine in South Asia. These are attributable to *V. negundo*’s rich variety of flavonoids and flavonoid glycosides, which include luteolin-7-glucoside (Figure 3), a compound that exhibits anti-oxidant activity and prevents selenite toxicity and cataractogenesis [31,72]. However, further study is required in order to fully elucidate its efficacy against DR.

#### 2.2.31. *Zea mays* L.

*Z. mays* L. (purple waxy corn) is easy to find anywhere in the world. *Z. mays* is an important source of anthocyanins, and of phenolic acids including *p*-coumaric acid (Figure 3), vanillic acid, protocatechuic acid, and flavonoids like quercetin and hesperetin. Traditionally, Native Americans have used it to treat a wide range of ailments. It makes an effective poultice, which can be used to treat bruises, swellings, sores, boils, and similar conditions. Chickasaw Indians treated itching skin by burning old corncobs and holding the affected part over the smoke. Recently, a study conducted to explore the combined anti-cataract and anti-retinopathy activities of Z. mays and ginger revealed suppression of both aldose reductase’s inhibitory activity, and of OS. These changes effectively delayed cataractogenesis in diabetes, and increased the number of neurons in the ganglionic cell layer was present. Additionally, the thickness of total retina and of the retinal nuclear layer were found to be increased in diabetic rats. These studies have proven the capability of this two-functional food against DR [73].

### 2.3. Anti-diabetic Effects of Terpenoid and Steroid Phytochemicals

#### 2.3.1. *Alpinia zerumbet*

*A. zerumbet* is widely distributed in subtropical and tropical regions. The secondary metabolites found in its roots inhibit AGE formation. Labdadiene (Figure 3), a triterpenoid bioactive compound, inhibits the formation of α-dicarbonyl compounds and fructosamine, both of which are important causative factors in AGE formation, and thereby reduces the occurrence of diabetic retinopathy [74]. While the potency of labdadiene against DR is clear, there remains a need to conduct further trials using in vitro and in vivo animal models.

#### 2.3.2. *Andrographis paniculata* Nees

*A. paniculata* nees is widely distributed and cultivated in Southern Asia, China, and Europe. Andrographolide (Figure 3), a major bioactive compound isolated from this plant possesses anti-inflammatory activity, which makes it useful for treating inflammatory bowel disease, pulmonary fibrosis, and cigarette smoke-induced lung injury. It also possesses anti-angiogenic effects and inhibits DR. In STZ-induced diabetic mice, it reduced the retinal vessel numbers and decreased elevated VEGF levels via binding KDR or FLT1 receptors. It also lowered DR via an anti-inflammatory mechanism, as retinal inflammation is critically involved in the progression of DR. It has been shown to inhibit the expression of inflammatory cytokines such as TNF-α, IL-1β, and IL-6; reduce leukostasis; and to decrease BRB breakage during DR development. In addition, it inhibits the activation of an NF-κB signaling pathway by decreasing the increased nuclear translocation and phosphorylation of NF-*κ*B, p65, kappa-B, and kappa-B kinase. Furthermore, it inhibits the nuclear translocation of Egr1 along with related downstream genes such as serpentine 1, PAI-1, and a transmembrane glycoprotein, which is involved in the progression of DR. Ultimately, andrographolide is a promising compound for the treatment of DR, which functions by inhibiting activated VEGF, NF-*κ*B, and early growth response 1 (EGR1) signaling pathways [75]. However, its toxicity is not well understood, and clinical trials in experimental animal models are required.

#### 2.3.3. *Astragalus membranaceous*

*A. membranaceous* possesses astragaloside IV, a novel saponin, is the major active compound isolated from this plant. It ameliorates DR via inhibition of AGE formation, hepatic glycogen phosphorylase, and glucose-6 phosphate activities, oxidative stress, and activation of NF-κB in STZ-induced diabetic mice. Its mechanism of action includes inhibition of ERK1/2 phosphorylation, NF-κB activation, RGCs dysfunction, which provides protection against retinal neurodegeneration. It also inhibits the activation of AR and downregulates the expression of VEGF, TNF-α, IL-1, IL-6, and MMPs. Astragaloside IV (Figure 3) is a good potential source of antidiabetic agents further investigated of its clinical efficacy may prove fruitful for the pharmaceutical industry [76]. *Astragalus* polysaccharides (APS), bioactive components of this plant lower the IL-1, 6, -12, and IL-1β, iNOS, and INF-gamma and increase the IL-4, -5, -10, and TGF-β. All this reveals that bioactive components of *A. membranaceous* have a potential antidiabetic effect and its related complications.

#### 2.3.4. *Origanum majorana* L.

*O. majorana***,** an herbaceous and perennial plant, is native to Southern Europe and the Mediterranean. It was considered a symbol of love or happiness by the ancient Greeks. *O. majorana* extracts exhibit anti-AGE formation, antiglycation, and anti-oxidant activities. It also promotes the major mechanism underlying inhibition of AGE formation, inhibiting of conversion of dicarbonyl intermediates, reducing sugars, and amadori adducts to AGE [77]. The antiglycative effects of triterpenoids ursolic acid (UA) (Figure 3) and oleanolic acid (OA) (Figure 3), which are major bioactive compounds present in this plant, have been studied in terms of their ability to attenuate DR. Both potentially reduce renal sorbitol dehydrogenase activity, with OL promoting renal glyoxalase I activity, increasing renal GLI mRNA expression, and reducing renal methylglyoxal levels [78]. Furthermore, experimental studies in animals on the protective effect and mechanism of UA have revealed that UA suppresses the vascularization, reduces the VEGF, COX-2, and MMP-2 expression in renal tissue, and promotes recovery from DR [79]. All this indicate that supplementation with UA or OA may help in alleviating the DR.

#### 2.3.5. *Panax quinquefolius*

*P. quinquefolius* is commonly known as ginseng and is valued for its bioactive ginsenosides, which are protopanaxatriol derivatives. North American ginseng’s effectiveness in amelioration of DR and cardiopathy is accomplished through its antioxidant and antihyperglycemic properties, and it has gained recognition as an inexpensive means of treating diabetic complications [80]. Ginsenoside-Rb1 (Figure 3), a bioactive terpenoid glycoside, is isolated from the *Panax* ginseng root, and has a long history of medicinal use. It suppresses VEGF release from retinal pigment epithelial cells and prevents the progression of DR [81]. Ginsenoside-Rg3 is another *Panax*-derived bioactive triterpenoid saponin which has been evaluated in retinal cells of diabetic rats, Where it was found to downregulate VEGF and TNF-α expression, ultimately ameliorating DR [82].

#### 2.3.6. *Zingiber zerumbet*

*Z. zerumbet* is well distributed throughout tropical and subtropical areas of South East Asia. Zerumbone (Figure 3), a sesquiterpene isolated from the rhizome of several species of genus *Zingiber*, is mainly used as food flavoring agent but also possesses a range of anti-tumor, anti-inflammatory, anti-oxidant, and anti-proliferative properties. It can also reduce the effects of nutritional steatohepatitis by regulating the causative genes involved in oxidative, inflammatory and fibrogenesis processes. It reduced high sugar levels and ameliorated diabetes by inhibiting pro-inflammatory cytokines, chemokines, and regulatory genes in the kidneys of diabetic mice. Additionally, it blocks AGE formation and provides protection against retinal damage induced by hyperglycemia. A further mode of action involves suppression of retinal permeability, which in turn decreases vascular permeability and inhibits BRB breakdown. BRB breakdown is an important causative factor in DR progression. It can further ameliorate DR through its anti-inflammatory activity by inhibiting the NF-κB activation signaling pathway and blocking degradation and phosphorylation of kappa-B. Finally, it blocks the p38 MAPK activation and AGE/RAGE signaling pathways in the retina of diabetic mice, providing an additional protective effect. Owing to its ability to hit multiple DR-related targets, zerumbone plays an important role in the recovery of DR and can be utilized as an adjunct therapy for treating diabetic microvascular complications [83].

### 2.4. Anti-diabetic Effects of Alkaloid Phytochemicals

#### *Cnidium* *officinale*

*C. officinale* contain butylidenephthalide (BP), an alkaloid phthalide, is isolated from its volatile oil and possesses antiangiogenic and other pharmacological properties that play a role in p38 and ERK1/2 signaling pathway activation, exhibiting anti-platelet, anti-inflammatory, anti-anginal, and anti-atherosclerotic activities. Extracts from *C. officinale* and BP inhibit AGE, RAGE, VEGF, and DLL4 expression and exert antiangiogenic effects on retinal neovascularization in-vitro and in-vivo experimental models, with commensurate inhibition of DR [84].

### 2.5. Anti-diabetic Effect of Other Phytochemicals

#### 2.5.1. *Lycium barbarum*

This plant is commonly known as Goji- or wolf berry. This plant is famous in Chinese medicine and has a variety of pharmacological effects due to the presence of valuable phytochemicals including zeaxanthin, betaine, carotene, β-sitosterol, polysaccharide, and several vitamins. *L. barbarum*’s anti-apoptotic properties make it a potential source of therapeutics for the treatment of DR. In retinal cells, plant-derived polysaccharides prevent oxidative stress-induced cell apoptosis by up-regulation of anti-apoptotic gene Bcl-2 and downregulation the pro-apoptotic gene BAX. Taurine, a non-essential free amino acid isolated from *L. barbarum*, reverses diabetes and cell apoptosis via multiple mechanisms; it inhibits caspase-3 activity, PPAR receptor activation, and downregulation of VEGF mRNA expression. Neuronal and retinal pigment epithelium cells are an important part of the blood-brain barrier that prevents apoptosis and helps in maintaining the integrity of BRB. This ameliorates inflammation and angiogenesis, subsequently preventing retinal tissue damage and loss of vision. These findings show that *L. barbarum* is effective against diabetic retinopathy by means of an anti-inflammatory, antiapoptotic, and anti-angiogenic mechanism [62,85]. Experiments in the diabetic retina of rats indicate that chronic turaine supplementation lowers the level of glutamate, γ-aminobutyric acid, intermediate filament glial fibrillary acidic protein, and *N*-methyl-d-aspartate receptor subunit 1, and elevates glutamate transporter expression, which is followed by amelioration of DR. This mechanism is known as anti-excitotoxicity of glutamate in rats [86]. Additional studies on the protective effect of taurine indicate that it possesses an anti-diabetic effect, prevents loss of body weight, and minimizes electrophysiological changes in the retina of diabetic animals [87].

#### 2.5.2. *Paenonia lactiflora*

*P. lactiflora* is widely distributed throughout China. It is mainly used for the ornamental purposes. Peoniflorin, a monoterpene glucoside, is the principal bioactive component isolated from the root of this plant and effective against rheumatoid arthritis, mesenteric hyperplastic nephritis, systemic lupus erythematosus, and hepatitis. It attenuates diabetic retinopathy by alleviating retinal inflammation through upregulation of SOCS3 expression, suppression of toll-like receptor 4 (TLR4) signaling, and reduction of MMP-9 production, and of inflammatory factors including TLR4/p38/NF-κB signaling pathways in BV2 cells [88]. Aminoguanidine (Figure 3) is a nucleophilic hydrazine compound that suppresses glycation and prevents AGE formation with commensurate inhibition of DR [89].

#### 2.5.3. *Scutellaria barbata*

In 2017, Zhoou determined that treatment of STZ-induced diabetic mice with *S. barbata* reversed BRB breakdown and decreased expression of tight junction proteins including claudin-1, and -19. Moreover, it diminished mRNA expression of TNF-α, IL-1β, ICAM-1, and NF-κB [90]. These findings indicated that *S. barbata* reverses DR by preventing retinal inflammation and lowering expression of tight junction proteins. As with other medicinal plants, there remains a need to explore in detail the mechanism(s) by which *S. barbata* phytochemicals ameliorate DR.

## 3. Discussion

Natural products are a rich source of bioactive compounds with potential activity against several diseases and metabolic ailments, including oxidative stress-induced diseases, gastrointestinal tract infections, inflammatory diseases, diabetes, cancers, and drug-induced nephro- and hepatotoxicity (Table 1). DM is associated with several complications, including DR, in which cellular damage occurs to the retina, causing impaired vision or total loss of vision. Importantly, a variety of population-based studies have revealed that diabetic complications such as DR, diabetic nephropathy, and diabetic cardiomyopathy are interlinked, and share common pathways [91,92]. Current therapies for these conditions include anti-VEGF injections [93], laser photocoagulation, vitreoretinal surgery, and administration of steroid agents [94]. Although, these therapies are largely effective but have certain limitations. For example, majority of world’s population have severely limited access to affordable drugs and specialized care [93]. In addition to this, serious adverse effects to existing treatments have been reported. For example, anti-VEGF injections have a short duration of action, and may trigger retinal detachment in patients with pre-existing preretinal fibrosis [95].

Laser photocoagulation can destroy and burn part of retina, resulting in permanent loss of vision. Difluprednate, a corticosteroid used in eye drops, reduces inflammation and pain in patients who have undergone eye surgery, but may temporarily cause blurred vision, and prolonged use of this medication may result in fungal infection [96]. Furthermore, multiple factors are involved in DR progression, including VEGF, IL-6, IL-α, TNF-α, AGE, NF-κB, ROS, and many others are involved in signaling pathway regulation. Considering these factors, there is a need to find multitargeted and cost-effective therapies, which can easily be made available to the majority of world’s population. Medicinal plants contain a large variety of biologically active ingredients that have the ability to act on multiple pharmaceutical targets at a time. By hitting multiple targets, recovery from DR is faster, which potentially saves lives, time, and money. In addition, the use of phytomedicine in combination with laser or surgical treatments can dramatically lower prognosis, improving vision and reversing the effects of diabetes. Therefore, medicinal plants are recommended as an innovative approach and best-choice of treatment for diabetes-related complications including DR.

In spite of various beneficial properties associated with the use of medicinal plants, several factors need to be considered with regard to their safe use and quality standards. Standardized procedures should be taken into consideration when producing natural products, and also when using them in preclinical and clinical experiments. While the majority of herbal medicines are easy to acquire, as there is no requirement for a prescription from an authorized health care practitioner or physician. In addition, consistent quality, effectiveness, and safety are not guaranteed as these medicines are not formally analyzed for toxicity or quality. Therefore, standardization and quality control should be a high priority for improving their efficacy and consistency. In addition, while some natural products exert powerful in vitro effects, they may not be effective in vivo, for a variety of reasons including poor pharmacokinetic properties such as poor absorption and low bioavailability. Therefore, there is an additional need to develop advanced procedures for improving both their pharmacokinetic and pharmacodynamic characteristics. All these considerations recommend that relevant checkpoints and supervisory authorities should be established to improve public health with suitable standards and safety criteria where herbal medicines are concerned.

Medicinal plants have emerged as an excellent source of therapeutically-active compounds, which have been shown to have a positive effect against diabetes and diabetes-related complications. A variety of non-nutrient secondary metabolites, including phenolics, alkaloids, and terpenoids, are isolated from different parts of plants that can modulate the cell signaling pathway involved in the pathogenesis of DR . Evidence from numerous studies suggests that phytochemicals exhibit their effects after crossing through the blood-retinal barrier and ultimately provide protection against the development and progression of diabetic retinopathy as shown in Table 1.

On the basis of above literature review, a variety of recommendations may be put forth. Several plants have been evaluated in terms of their efficacy against aldose reductase activity only. These include, for example, *A. distichum*, *A. marmelos*, *P. frutescens*, *S. hexaphylla*, and *T. purpurea* [23,24,54,70]. Therefore, detailed studies should be conducted to explore multiply-targeted signaling pathways affected by these medicinal plants and their bioactive compounds using a variety of in vitro and in vivo experimental models. Few medicinal plants have been studied in terms of their antioxidant activity-related effectiveness against DR. Those that have include *J. regia* and *V. negundo* [31,42,72]. However, these plants and their phytoactive extracts still require detailed investigation into the mechanisms by which they affect signaling pathways. Inhibition of AGE formation is the most common means by which DR may be prevented, treated and cured, and much research has been undertaken in order to identify medicinal plants, and their phytochemicals, which have activity as AGE formation inhibitors. A number of species have been identified, including *A. koraiensis*, *P. multiflorum*, *P. vulgaris* and *A. zerumbet.* However, signaling pathways for many medicinal plants and their phytochemicals still need to be investigated, for example, *L. japonica*, *M. officinalis*, *M. oleifera*, *M. albla*, 5′-methoxybiphenyl-3,4,3′-triol from *O. schwerinae*, *S. miltiorrhiza*, *S. hexaphylla*, *Z. mays* L, *A. paniculata*, *P. quinquefolius*, *C. officinale*, and *P. lactiflora.* Various plants extracts have been studied, and their potency against DR demonstrated, for example those from *A. pilosa*, *G. uralensi*, J. *regia*, *M. albla*, *C. officinale*, *L. barbarum*, and *S. barbata*, but as yet no one bioactive ingredient has been pointed out as a specific blocking agent for DR. Therefore, there is an urgent need to unveil the main bioactive compound, or combination of compounds, responsible for inhibiting DR. Many plants exhibit potential pharmacological activities and antidiabetic effects, but thus far no standardization and toxicological experiments have been carried out using the active ingredient as a biomarker. For example, while *C. religiosum* is commonly employed to treat jaundice, syphilis, and gonorrhea, and is a source of two bioactive constituents, namely isorhamnetin and myricetin (Stefek [28]), and *D. chrysotoxum* is a rich source potential agents such as gigantol and syringic acid, having antihyperglycemic, antioxidant, immunomodulatory, and anti-tumor effects [36], no standardization study aimed at reliably getting high-quality extracts from these plants has been performed to date. Fruit pericarp *L. Chinensis*, usually wasted after eating the fruit, is a rich source of polyphenols and tannins, having potential ethnopharmacological anti-tussive, antipyretic, analgesic, and diuretic properties, could be utilized as a source of antidiabetic agents in different pharmaceutical industries [43]. Therefore, attention should also be given to standardization of suitable techniques for getting quality extracts from *L. chinenesis*. Additionally, *L. lucidum*, *L. japonicae*, *M. officinalis*, *C. edulis*, and *M. albla* extraction methods also require standardization. Some plants, including *A. distichum*, *A. marmelos*, *A. pilosa*, and *Z. zerumbet*, have demonstrated activity against DR in in vitro studies. However, to date no study has been conducted to establish their potency in in vivo. Therefore, such a study should be undertaken within the near future. Various medicinal plants and their phytochemicals demonstrate potential ethnopharmacological effects and their ability to block DR has been studied in detail. These species include *C. religiosum*, *D. chrysotoxum*, *G. biloba*, *P. grandiflorum*, *P. cuspidatum*, *P. lobate*, *S. miltiorrhiza*, *T. catappa*, and *A. membranaceous.* Some of these have been proven to be effective in the treatment of DR in clinical trials. *P. grandiflorum*, for example, demonstrated anti-obesity, anticancer, antiallergy, and neuroprotective effects, with decoctions proving effective in the treatment of patients with DR [56]. *S. miltiorrhiza*, similarly, is anti-inflammatory, provides vascular endothelial protection, and has anti-pulmonary fibrosis effects. Clinical trials have revealed that this plant contributes to improved vision in DR patients [66]. *G. biloba* has been used for many years due to its pharmacological activities against a variety of diseases and has anticancer, anti-platelet activation, neuroprotective, and antioxidant properties in addition to its antidiabetic effects. Successful clinical trials have been conducted in which *G. biloba* was used as a supplement to cure treat DR patients [39,40].

A variety of edible medicinal plants i.e., *A. marmelos*, *A. paniculate*, *A. koraiensis*, *C. nitidissima*, *C. edulis*, *D. chrysotoxum*, *G. uralensi*, *L. chinensis*, *L. japonia*, *L. barbarum*, *M. officinalis*, *M. oleifera*, *M. alba*, *O. majorana*, *O. schwerinae*, *P. frutescens*, *P. grandiflorum*, *P. odoratum*, *P. cuspidatum*, *P. vulgaris*, *S. miltiorrhiza*, *S. hexaphylla*, *Z. mays*, and *Z. zerumbet* are routinely consumed across different regions of the world [30,33,97,98,99]. These edible plants are a source of food as well as medicine. Therefore, regular consumption as part of a daily diet may form part of a first-line therapy in the treatment of DR.

Above literature review reveals that no study has yet been conducted specifically targeting the JAK/STAT signaling pathway, an important mechanism involved in DR. Consequently, research should be carried out to explore the active compounds targeting this pathway. In addition, the hexosamine pathway is another important signaling pathway but few studies investigating its role in the progression of DR have been conducted in recent years.

**Figure 4:**
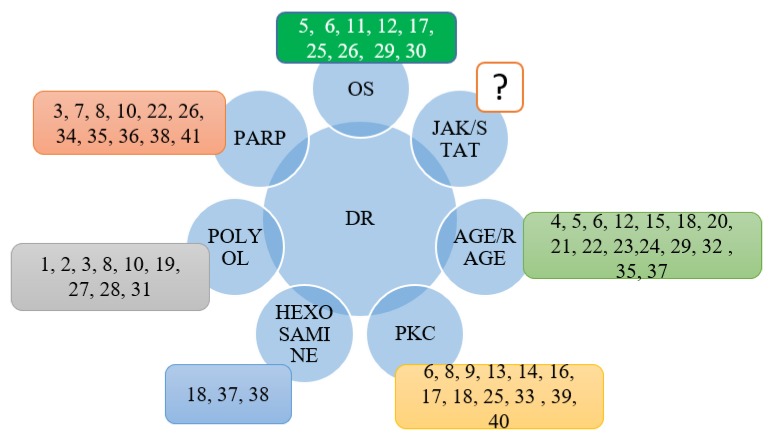
**1**. *Abeliophyllum distichum*; **2**. *Aegle marmelos*; **3**. *Agrimonia pilosa* ledeb; **4**. *Aster Koraiensis*; **5**. *Camellia nitidissima* Chi; **6**. *Carpobrotus edulis*; **7**. *Cochlospermum religiosum*; **8**. *Dendrobium chrysotoxum*; **9**. *Ginkgo biloba*; **10**. *Glycyrrhiza uralensi*; **11**. *Juglans regia* L.; **12**. *Litchi chinenesis*; **13**. *Ligustrum lucidum* Ait; **14**. *Lonicerae japonicae* Flos; **15**. *Melissa officinalis*; **16**. *Morniga oleifera* Lam; **17**. *Morus alba*; **18**. *Osteomeles schwerinae* C.K. Schneid; 19. *Perilla frutescens*; **20**. *Platycodon grandiflorum*; **21**. *Polygonatum odoratum*; **22**. *Polygonum cuspidatum*; **23**. *Polygonum multiflorum*; **24**. *Prunella vulgaris*; **25**. *Pueraria lobata*; **26**. *Salvia miltiorrhiza* Bge; **27**. *Stauntonia hexaphylla*; **28**. *Tephrosia purpurea*; **29**. *Terminalia catappa*; **30**. *Vitex negundo*; **31**. *Zea mays* L.; **32**. *Alpinia zerumbet*; **33**. *Andrographis paniculata* Nees; **34**. *Astragalus membranaceous*; **35**. *Origanum majorana* L.; **36**. *Panax quinquefolius*; **37**. *Zingiber zerumbet*; **38**. *Cnidium Officinale*; **39**. *Lycium barbarum*; **40**. *Paenonia lactiflora*.

Phytochemicals controlling DR though several signaling pathways provide hope for the development of safe and effective therapies to reverse diabetes-related vision impairment (Figure 4). In addition, controlling and deactivation of PKC, hexosamine, polyol, PARP, and OS signaling pathway can block the progression of several diabetes-related complications. It has been suggested that it is good to slow down or block the DR progression pharmacologically for development of new disease treatments. In DR, AGE formation and OS contribute to retinal tissue damage and loss of vision. We propose that nutritional phytochemicals, which act through several signaling pathways, should be deeply explored in clinical trials and preclinical animal models. In addition, based on their chemical structure, derivatives of potent natural drugs may by synthesized using a structure/activity modelling procedure.

## 4. Materials and Methods

A broad-spectrum study was conducted using published English works in recent five years in various electronic databases including Science Direct, PubMed, Scopus, Google Scholar, and Embase. We investigated for medicinal plants along with their bioactive phytochemicals exhibiting their role in attenuating DR focused on the target based mechanism.

## 5. Conclusions

The scientific works discussed here provide evidence of medicinal plant use, either as the source of individual extracted compounds, or as a mixture of various bioactive components, which demonstrate remarkable suppression of cellular damage to the retina, or the improvement of vision in general. Use of synthetic retinoprotective agents or therapies is limited due to their cost and availability. Therefore, natural products, which contain diverse and potent active agents, and are inexpensive and easily-available, should be prioritized as a means for restoring human health. Furthermore, the addition of medicinal herbs in daily diets can reduce the risk of diabetes and diabetes-related complications, including DR, and significantly improve human health.

## Figures and Tables

**Table 1:** Phytochemicals in diabetic retinopathy.

Sr. No	Plant Name	Active Constituent	Traditional Use	Family	Class	Pharmacological Target	Pharmacological Activity	Reference
1	*Abeliophyllum distichum*	Acteoside		Oleaceae	Phenolic	Aldose reductase	Anti-hypertensive Anti-diabetic Anti-inflammatory Anti-cancer	[23]
2	*Aegle marmelos*	Cinnamic acid	Diabetes mellitus Ulcer Beriberi Cholera Ophthalmia	Rutaceae	Phenolic acid	Aldose reductase	Anti-inflammatory Analgesic Anti-pyretic Anti-hyperglycemic Anti-diarrheal Anti-microfilarial	[24]
3	*Agrimonia pilosa ledeb*	Agrimoniin	Abdominal pain Heat stroke Headache Sore throat	Rosaceae	Phenolic	Aldose reductase PPAR-γ Glucose transporter type 4	Anti-cancer Anti-oxidant Anti-allergy Anti-inflammatory Anti-nociception	[26]
4	*Alpinia zerumbet*	Labdadiene	Anti-inflammatory Ati-fungal Anti-bacterial	Zingiberaceae	Triterpenoid	AGE	Anti-hypertensive Anti-ulcerogenic Diuretic Sedative	[74]
5	*Andrographis paniculata*	Andrographolide	Cooling effect Detoxification Sore throat Respiratory tract infection	Acanthaceae	Diterpenoid	TNF-α IL-1β IL-6 NF-κB PAI-1 EGR1	Hepatoprotective Anti-oxidant Anti-hyperglycemic Anti-cancer Anti-platelet aggregation	[75]
6	*Aster Koraiensis*	Chlorogenic acid 3,5,di-caffeoylquinic acid	Diabetes Pertussis Chronic bronchitis Pneumonia	Asteraceae	Phenolic acid	AGE NF-κB	Anti-diabetic Anti-apoptosis	[27]
7	*Astragalus membranaceous*	Astragaloside IV	Stomach ulcer Diabetes Diuretics Tonic Fever	Leguminosae	Triterpenoid	NF-κB VEGF TNF-α IL-1 IL-4 IL-5 IL-10 IL-6 MMPs IL-1β iNOS TGF-β	Immunomodulatory Anti-apoptosis Anti-inflammatory	[76]
8	*Camellia nitidissima*		Cancer Diarrhea Sore throat High blood pressure Irregular menstruation	Theaceae	Flavonoids	AGE OS	Anti-oxidant	[33]
9	*Carpobrotus edulis*	Ellagic acid	Throat infection Tuberculosis Diarrhea Dysentery Mouth ulcer Stomach ailments	Aizoaceae	Phenolic acid	AGE OS GAP VEGF Bax HIF-1α	Anti-bacterial Anti-oxidant Anti-proteus Anti-microbial	[30]
10	*Cnidium officinale*	Butylidenephthalide	Inflammation High blood pressure Menstrual pain	Umbelliferae	Alkaloid	ERK1/2 AGE/RAGE VEGF DLL4	Larvicidal Acaricidal Anti-hyperglycemic Anti-angiogenic Anti-inflammatory	[84]
11	*Cochlospermum religiosum*	Isorhamnetin	Sedative Jaundice Gonorrhea Syphilis Stomach ailments	Cochlospermaceae	Flavonoid	RAGE-Src-ERK1/2-FAK-1 paxillin signaling pathway	Anti-oxidant Anti-microbial Immunomodulatory	[28]
12	*Dendrobium chrysotoxum*	Gigantol Syringic acid	Moisten and nourish skin Longevity Tuberculosis Anorexia Eye sight	Orichidaceae	Phenolic	VEGF MMP2/9 IL-1β, IL-3, IL-6, IL-10, IL-12 IκB ICAM-1	Anti-angiogenesis Anti-inflammatory Anti-oxidant Anti-hyperglycemic Immunomodulatory	[36]
13	*Ginkgo biloba*		Blood disorders	Ginkgoaceae	Flavonoid	TNF-α VEGF	Anti-angiogenesis Anti-oxidant Anti-inflammatory Neuroprotective Hepatoprotective Anti-stress	[40]
14	*Glycyrrhiza uralensi*		Hepatitis C Peptic ulcer Diabetes Skin diseases Pulmonary diseases	Fabaceae	Flavonoid	PPAR-γ	Anti-depressant Anti-oxidant Immunomodulatory Hepatoprotective Anti-viral Anti-inflammatory Hepatoprotective Anti-cancer	[41]
15	*Juglans regia*		Diabetes Inflammation Infection		Flavonoid Phenolic	PARP OS COX-2 Caspase-3	Anti-oxidant Anti-microbial Sedative Anti-hyperglycemic	[42]
16	*Litchi chinenesis*		Diabetes Obesity Epigastric pain Herniae-like conditions Neuralgic apin	Sapindaceae	Polyphenol	AGE OS	Anti-inflammatory Ant-ioxidant Hepatoprotective	[43]
17	*Ligustrum lucidum*	Specnuezhenide	Eyesight Dizziness Fever Insomnia Cancer	Oleaceae	Phenolic	HIF-1α VEGF	Anti-angiogenesis Hepatoprotective Anti-diabetic	[44]
18	*Lonicerae japonicae*	Chlorogenic acid	Inflammation Headache Acute fever Eye sight Heat stroke	Caprifoliaceae	Phenolic	VEGF	anti-angiogenesis anti-nociceptive anti-inflammatory analgesic anti-bacterial	[47]
19	*Lycium barbarum*	Taurine	Blurry vision Abdominal pain Infertility Dry cough Fatigue Dizziness Headache	Solanceae	Amino acid	Bcl-2 Bax Caspase-3 PPAR VEGF	Anti-apoptosis Anti-angiogenesis Immunomodulator Anti-aging Neuroprotective	[87]
20	*Melissa officinalis*	Rosmarinic acid	Indigestion Cardiac failure Anemia	Lamiaceae	Phenolic acid	AGE	Anti-angiogenesis Anti-cancer Anti-oxidant Neurotropic Anti-microbial Anti-bacterial	[48]
21	*Moringa oleifera*		Culinary use Malnutrition	Mornigaceae	Polyphenolic	TNF-α IL-1β VEGF PKC-β	Hypolipidemic Anti-atherosclerosis Hypocholesterolemic Anti-angiogenesis Anti-inflammatory Anti-oxidant	[51]
22	*Morus albla*		Feedstock for silkworms Constipation Diabetes	Moraceae	Phenolic	PKC OS Caspase-3 VEGF Bax Bcl-2	Anti-apoptosis Anti-angiogenesis Antioxidant Anti-microbial Hypoglycemic hepatoprotective Anti-inflammatory	[53]
23	*Origanum majorana* L.	Ursolic acid Oleanolic acid	Disinfectant Headache Indigestion Rheumatism Insomnia Diabetes Asthma Cataract Nervousness	Lamiaceae	Triterpenoid	AGE COX-2 MMP-2 OS	Anti-oxidant Anti-hyperglycemic Anti-microbial Anti-proliferative Anti-cholinesterase	[79]
24	*Osteomeles schwerinae*	5′-methoxybiphenyl-3,4,3′-triol	Diarrhea Sore throat Arthritis Dysentery	Rosaceae	Phenolic	Aldose reductase VEGF FGF-2 IGFBPS PAI-1	Anti-diabetic Anti-angiogenesis Anti-oxidant	[33]
25	*Paenonia lactiflora*	Aminoguanidine	Rheumatoid arthritis Systemic lupus erythematous Fever Spasm Muscles cramping Dysmenorrhea	Paeoniaceae		TLR4 MMP-9 NF-*κ*B	Anti-inflammatory Anti-oxidant Anti-thrombosis	[88]
26	*Panax quinquefolius*	Ginsenoside-Rb1	Aphrodisiac Restorative Nootropic Antiaging Tonic	Araliaceae	Steroid glycoside	VEGF TNF-α	Antioxidant Anti-angiogenesis Antidiabetic Anti-coagulant	[81]
27	*Perilla frutescens*	Rosmarinic acid	Cough Bacterial infection Fungal infection Allergy Tumor Intestinal disorder	Lamiaceae	Phenolic acid	p21WAF1	Anti-angiogenesis Anticancer Anti-inflammatory Anti-allergy Anti-depressant Anti-allergy	[54]
28	*Platycodon grandiflorum*	Luteolin	Cough Inflammation Fever	Cmpanulaceae	Flavonoid	Aldose reductase	Anti-oxidant Anti-cancer Hepatoprotective Anti-hyperlipidemia	[56]
29	*Polygonatum odoratum*		Diabetes	Liliaceae	Flavonoid	AGE	Antihyperglycemic Antioxidant	[57]
30	*Polygonum cuspidatum*	Polydatin Resveratrol Emodin glucopyranoside Emodin	Allergy Diabetes	Polygonaceae	Phenol	HMGB1 AGE NF-κB IL-4 PPAR-α/-β	Anti-AGE formation Anti-inflammatory Anti-oxidant Anti-bacterial Anti-apoptosis	[57]
31	*Polygonum multiflorum*		Anti-aging Tonic	Polygonaceae	Phenolic Flavonoid	AGE	Neuroprotective Anti-oxidant Myocardial protective Anti-inflammatory	[60]
32	*Prunella vulgaris*		Headache Goiter Cancer High blood pressure Lymphatic system disorder	Lamiaceae	Phenolic Flavonoid	Aldose reductase AGE OS	Anti-oxidant Immunostimulatory Anti-HIV Anti-allergy Anti-inflammatory	[61]
33	*Pueraria lobata*	Puerarin	Neuro-protective Hepato-protective Analgesic inflammation Fever	Fabaceae	Flavone	iNOS IL-1β ICAM HIF-1α VEGF Bax Caspase-3 TNF-α ERK p38 MAPK	Anti-apoptosis Anti-angiogenesis Antioxidant Vasodilatory Neuroprotective Hepatoprotective Anti-pyretic Analgesic Anti-inflammatory	[64]
34	*Salvia miltiorrhiza*		Coronary heart disease Cerebrovascular	Labiatae	Phenolic	Lp-PLA2 IL-1 IL-6	Antihyperglycemic Anti-arrhythmic Anti-pulmonary fibrosis Anti-inflammatory	[68]
35	*Scutellaria barbata*		Toxicity Heat relief Blood circulation promoter Pain and swelling	Lamiaceae		TNF-α IL-1β ICAM NF-κB	Anti-cancer Anti-leukemic	[90]
36	*Stauntonia hexaphylla*		Sedative Analgesic Diuretic	Lardizabalaceae	Flavonoid and phenolic	Aldose reductase	Anti-inflammatory Anti-HIV	[69]
37	*Tephrosia purpurea*		Ulcer Asthma Leprosy Cancer	Fabaceae	Flavonoid Phenolic	Aldose reductase	Anti-ulcer Anticarcinogenic Anti-lipidperoxidative Immunomodulator Anti-cancer	[70]
38	*Terminalia catappa*		Dermatitis Hepatitis Pyresis Diarrhea	Combretaceae	Tannin	LDL HDL	Anti-oxidant Anti-angiogenesis Anti-inflammatory Hepatoprotective Anti-diabetic Anti-bacterial Analgesic	[71]
39	*Vitex negundo*	Luteolin-7-glucoside	Eczema Ringworm Liver disorder Rheumatic pain Gout vermicide	Verbenaceae	Flavonoid	OS	Anti-oxidant Anti-inflammatory Analgesic Anti-microfilarial	[72]
40	*Zea mays*	Quercetin Coumaric acid	Diuretic Dysuria vasodilator Menorrhagia Nose bleeds	Poaceae	Flavonoid Phenolic	Aldose reductase OS	Anti-oxidant	[73]
41	*Zingiber zerumbet*	Zerumbone	Inflammation Toothache Fever Ingestion Diarrhea	Zingiberaceae	Sesquiterpene	NF-κB AGE/RAGE p38 MAPK	Anti-microbial Anti-nociceptive Anti-hyperglycemic Anti-inflammatory Anti-cancer Anti-allergy	[83]
